# Lactate dehydrogenase-to-lymphocyte ratio predicts overall and progression-free survival among patients with advanced small-cell lung cancer receiving first-line chemoimmunotherapy: A two-center retrospective observational study

**DOI:** 10.1097/MD.0000000000050249

**Published:** 2026-08-14

**Authors:** Tomofumi Hirose, Shuhei Teranishi, Nobuaki Kobayashi, Anna Tanaka, Yukihito Kajita, Ayami Kaneko, Masaki Yamamoto, Makoto Kudo, Takeshi Kaneko

**Affiliations:** a Respiratory Disease Center, Yokohama City University Medical Center, Yokohama, Japan; b Department of Thoracic Oncology, Kanagawa Cancer Center, Kanagawa, Japan; c Department of Pulmonology, Yokohama City University School of Medicine, Yokohama, Japan.

**Keywords:** immune checkpoint inhibitor, lactate dehydrogenase-to-lymphocyte ratio, overall survival, progression-free survival, small-cell lung cancer

## Abstract

Platinum plus etoposide combined with programmed death-ligand 1 (PD-L1) inhibitors is the standard first-line therapy for patients with advanced small-cell lung cancer (SCLC). However, reliable prognostic biomarkers are yet to be identified. The lactate dehydrogenase-to-lymphocyte ratio (LLR) is a potential indicator of tumor metabolism and host immune response; a high LLR is correlated with a poor prognosis in other carcinomas. The LLR may be a more comprehensive indicator of prognosis than the neutrophil-lymphocyte ratio (NLR) or platelet-lymphocyte ratio (PLR), which mainly reflect host immune status. This study evaluates the prognostic value of the LLR in patients with SCLC receiving first-line chemoimmunotherapy. This retrospective, two-center study was conducted at 2 university hospitals in Japan and analyzed 64 patients with extensive-stage SCLC or post-chemoradiotherapy recurrence of limited-stage SCLC who received platinum-etoposide plus a PD-L1 inhibitor as first-line therapy between August 2019 and December 2023. The LLR, NLR, and PLR were calculated from blood tests immediately before the start of first-line therapy, and cutoff values were set using the X-tile software. The prognostic significance of these markers was assessed using Kaplan–Meier survival analysis and the Cox proportional hazards model. Of the 64 patients included, 10 (16%) had high LLR (>471.5). The median duration of observation was 10.5 months (interquartile range: 6.0–19.0 months); by the time of data cutoff, 56 patients (87.5%) had experienced progressive disease and 36 (56.3%) had died. The high LLR group had significantly shorter overall survival (OS) and progression-free survival (PFS). Multivariate analysis was performed using only those factors (LLR/ECOG PS/bone metastasis regarding OS and LLR/bone metastasis regarding PFS) identified as significant in univariate analyses. A high LLR was an independent predictor of worse OS and PFS. While the NLR was an independent prognostic factor for OS, neither NLR nor PLR was a significant predictor of PFS. The LLR is a novel potential prognostic biomarker that integrates tumor metabolism and host immunity in patients with SCLC receiving first-line chemoimmunotherapy. Because it can be readily calculated from routine blood tests, it may serve as a cost-effective tool for risk stratification. However, further prospective validation in larger cohorts is warranted.

## 1. Introduction

Small-cell lung cancer (SCLC) accounts for approximately 15% of all lung cancers and is characterized by rapid progression and poor prognosis, with many patients presenting with distant metastases at diagnosis.^[1,2]^ Approximately 70% of patients with SCLC present with extensive-stage SCLC (ES-SCLC) at diagnosis.^[1]^ Platinum plus etoposide chemotherapy is the standard first-line treatment for ES-SCLC; however, its efficacy is limited.^[3]^ The addition of programmed death-ligand 1 (PD-L1) inhibitors, atezolizumab and durvalumab, to this regimen has improved survival in phase III trials,^[4,5]^ making chemoimmunotherapy the new standard. However, only a subset of patients benefit significantly, and early disease progression remains common.^[1,6]^ Given these circumstances, reliable biomarkers for predicting treatment response and survival are urgently required.^[6,7]^

PD-L1 expression in SCLC is generally low, and the tumor mutation burden has shown limited prognostic utility.^[7,8]^ Hence, blood-based biomarkers have gained attention owing to their accessibility and prognostic potential. In particular, lymphocytes play a central role in antitumor immunity, and peripheral blood lymphopenia is associated with impaired immune surveillance and increased systemic inflammation.^[9]^ Elevated neutrophil-lymphocyte ratios (NLRs) and platelet-lymphocyte ratios (PLRs), indicative of lymphocytopenia, have been identified as indicators of unfavorable prognoses.^[10,11]^ However, while these reflect host immunity, they do not reflect tumor aggressiveness.

Recently, the lactate dehydrogenase-to-lymphocyte ratio (LLR) has been reported to be a promising biomarker that reflects host immunity and tumor metabolism. Lactate dehydrogenase (LDH) is a key enzyme in glycolysis, and elevated LDH levels are associated with enhanced aerobic glycolysis, known as the Warburg effect, which plays a fundamental role in cancer progression.^[12,13]^ Additionally, lactate accumulation in the tumor microenvironment polarizes tumor-associated macrophages toward an immunosuppressive phenotype.^[14]^ The combination of low lymphocyte counts and high LDH in the LLR is a potential indicator of both tumor aggressiveness and host immune function. A high LLR has been shown to correlate with poor prognosis in diffuse large B-cell lymphoma and renal cell carcinoma.^[15,16]^

However, the role of the LLR as an independent prognostic marker in contemporary ES-SCLC treated with a combination of platinum, etoposide, and PD-L1 inhibitors is yet to be confirmed. Therefore, in this retrospective observational study, we aimed to assess whether the LLR is associated with the prognosis of patients with SCLC who were initially treated with a combination of platinum, etoposide, and a PD-L1 inhibitor. We also examined the relationships among prognosis, the NLR, and the PLR, which have been previously reported as prognostic factors.

## 2. Methods

### 2.1. Study design and patients

This retrospective observational study was conducted at 2 university hospitals in Kanagawa Prefecture, Japan: Yokohama City University Medical Center and Yokohama City University School of Medicine. The study procedures followed the guidelines set in the Declaration of Helsinki and received approval from the Ethics Committee at Yokohama City University (approval number: B191200044). As this was a retrospective study, informed consent was not required.

The research cohort consisted of individuals with SCLC who received a combination of chemotherapy and PD-L1 inhibitors as their initial treatment from August 2019 to December 2023. The criteria for patient eligibility included: diagnosis of SCLC confirmed through tissue or cell specimen analysis; diagnosis of ES-SCLC or post-chemoradiotherapy recurrence of limited-stage SCLC; and administration of either platinum plus etoposide plus atezolizumab or platinum plus etoposide plus durvalumab as the initial treatment from August 2019 to December 2023. Patients who had tumors with a mixture of histological types other than SCLC were excluded. All patients received carboplatin plus etoposide combined with either atezolizumab or durvalumab; no patients received cisplatin. Carboplatin was administered at an area under the curve of 4–5, etoposide at 60–100 mg/m^2^, atezolizumab at 1200 mg, and durvalumab at 1500 mg. Platinum plus etoposide combined with atezolizumab or durvalumab was administered for up to 4 cycles. Thereafter, maintenance therapy with atezolizumab or durvalumab was continued until disease progression or unacceptable adverse events. The following data were collected for each patient: age; sex; smoking history; Eastern Cooperative Oncology Group Performance Status (ECOG PS); cancer stage; presence of metastases in the brain, liver, or bones; and blood test results. The analysis employed an age threshold of 65 years.^[4,5]^ The ECOG PS was divided into 2 categories: good (0–1) and poor (2–4).^[4,5]^

### 2.2. Hematological data

We gathered information on LDH levels (U/L), absolute lymphocyte count (×10^9^/L), absolute neutrophil count (×10^9^/L), and platelet count (×10^9^/L) from blood tests conducted either the day before or on the day of first-line treatment initiation. Serum LDH levels were measured using the standardized method recommended by the International Federation of Clinical Chemistry and Laboratory Medicine. White blood cell counts were measured using a semiconductor laser-based flow cytometry method. Absolute lymphocyte counts, absolute neutrophil counts, and platelet counts were obtained from routine laboratory testing. We defined the LLR as the ratio of LDH to the absolute lymphocyte count, NLR as the ratio of the absolute neutrophil count to the absolute lymphocyte count, and PLR as the ratio of platelet count to the absolute lymphocyte count.^[10,11,15,16]^

### 2.3. Judging the efficacy of treatment

Tumor response was evaluated according to RECIST version 1.1.^[17]^ Progression-free survival (PFS) was calculated from the start of the initial treatment until either the disease advanced, the patient died, or the most recent follow-up (censored). Overall survival (OS) was calculated from the beginning of the initial treatment to either the patient’s death or the most recent follow-up, which was censored. The follow-up period concluded on August 30, 2024.

### 2.4. Statistical analysis

We established cutoff values for the LLR, NLR, and PLR in relation to OS, which is the most significant endpoint in cancer therapy, using X-tile 3.6.1 software.^[18]^ This software determines cutoff values using Kaplan–Meier log-rank chi-square statistics and can illustrate the strength of the association between biomarkers and outcomes. The Kaplan–Meier method was used to analyze the OS and PFS, and the log-rank test was used for assessment. Hazard ratios (HRs) and 95% confidence intervals (CIs) were calculated using the Cox proportional hazards model. A univariate analysis was conducted to identify the independent prognostic significance of the LLR, NLR, and PLR for OS and PFS. Factors that were significant in the univariate analyses were included in a multivariate analysis. Fisher’s exact test was used to analyze categorical variables, whereas the Mann-Whitney U-test was used to compare numerical data. Statistical significance was set at *P* < .05, and all statistical analyses were performed using a two-tailed approach. GraphPad Prism version 10 (GraphPad Software, San Diego, CA, USA) was used for data analysis along with EZR version 1.63 (Saitama Medical Centre, Jichi Medical University, Saitama, Japan).^[19]^

## 3. Results

### 3.1. Patient characteristics

Table 1 presents the demographic characteristics of the patients and summarizes the data collected from 64 individuals. The median age of the participants was 73 years (interquartile range: 66–76 years), with men constituting approximately two-thirds, and most having a history of smoking. More than 75% of the patients had a good ECOG PS (0–1), and none had an ECOG PS of 4. Seven patients (10.9%) experienced post-chemoradiotherapy recurrence of limited-stage SCLC, whereas the remaining patients were diagnosed with ES-SCLC. Brain and liver metastases were detected in 30% of patients, and bone metastases were found in 40%.

**Table 1:** Patient characteristics.

Characteristics	All patients (n = 64)	Low LLR (n = 54)	High LLR (n = 10)	*P*-value
Age (years)	73 (66–76)	73 (66–76)	73 (60–76)	.56
<65 years	10 (15.6)	7 (13.0)	3 (30.0)	
≥65 years	54 (84.4)	47 (87.0)	7 (70.0)	
Sex				>.99
Male	40 (62.5)	34 (63.0)	6 (60.0)	
Female	24 (37.5)	20 (37.0)	4 (40.0)	
Smoking status				>.99
Never	3 (4.7)	3 (5.6)	0	
Current or former	61 (95.3)	51 (94.4)	10 (100.0)	
ECOG PS				.04
0	12 (18.8)	12 (22.2)	0	
1	38 (59.4)	33 (61.1)	5 (50.0)	
2	10 (15.6)	7 (13.0)	3 (30.0)	
3	4 (6.3)	2 (3.7)	2 (20.0)	
4	0	0	0	
Stage				.58
Posttreatment recurrence of LS	7 (10.9)	7 (13.0)	0	
ES	57 (89.1)	47 (87.0)	10 (100.0)	
Programmed death-ligand 1 inhibitors				.73
Atezolizumab	42 (65.6)	36 (66.7)	6 (60.0)	
Durvalumab	22 (34.4)	18 (33.3)	4 (40.0)	
Brain metastasis				.45
No	46 (71.9)	40 (74.1)	6 (60.0)	
Yes	18 (28.1)	14 (25.9)	4 (40.0)	
Liver metastasis				.0001
No	42 (65.6)	41 (75.9)	1 (10.0)	
Yes	22 (34.4)	13 (24.1)	9 (90.0)	
Bone metastasis				.29
No	38 (59.4)	34 (63.0)	4 (40.0)	
Yes	26 (40.6)	20 (37.0)	6 (60.0)	
LLR	206.4 (128.8–334.3)	188.4 (120.7–259.2)	996.4 (814.0–1827.3)	<.0001
Low LLR (≤ 471.5)	54 (84.4)	54 (100.0)	0	
High LLR (> 471.5)	10 (15.6)	0	10 (100.0)	

Data are presented as numbers (%) or medians (interquartile ranges). Fisher’s exact test was used to compare the proportions of categorical data between the groups. The Mann–Whitney *U* test was used to compare numerical data between the groups.

ECOG PS = Eastern Cooperative Oncology Group Performance Status, ES = extensive stage, IQR = interquartile range, LLR = lactate dehydrogenase to lymphocyte ratio, LS = limited stage.

### 3.2. The LLR, NLR, and PLR cutoff values

**Figure 1.**
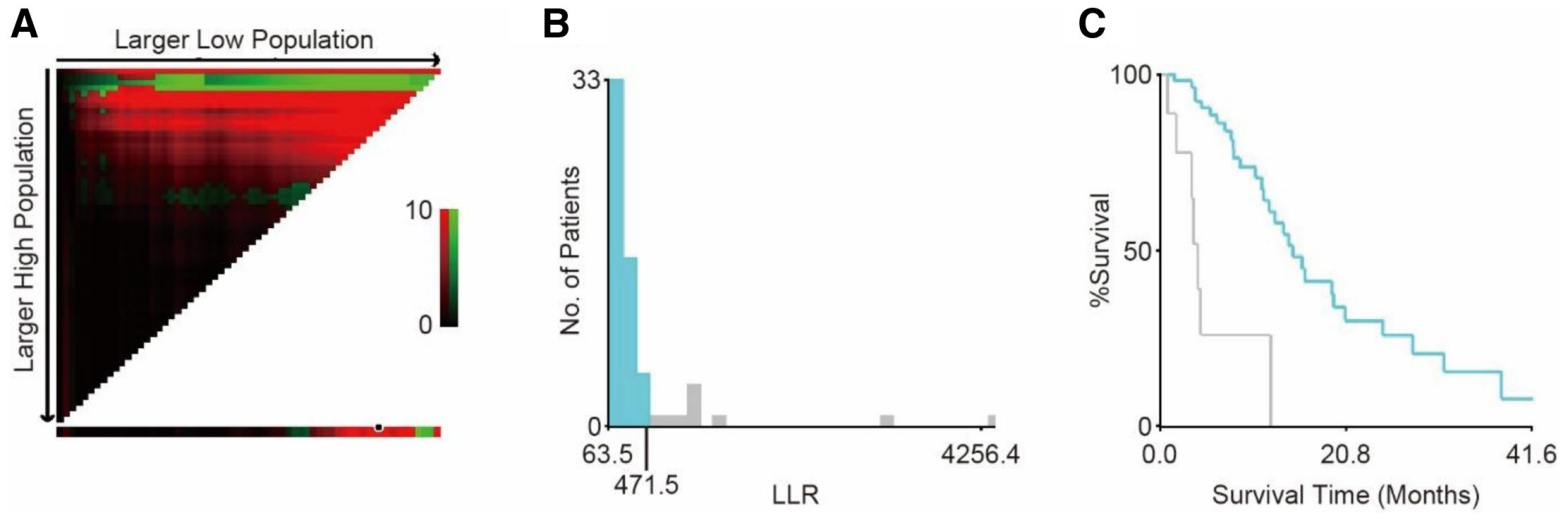
Cutoff value for the LLR determined using X-tile analysis. (A) X-tile plot for the LLR and overall survival. Each point in the plot represents a possible cutoff value, and the color intensity indicates the strength of the association between the LLR-based grouping and overall survival based on the log-rank chi-square statistic. The black/white circle indicates the optimal cutoff point selected by X-tile analysis. (B) Histogram showing the distribution of LLR values. Patients were divided into low- and high-LLR groups according to the optimal cutoff value of 471.5. (C) Kaplan–Meier survival curves for overall survival in patients with low and high LLR. LLR = lactate dehydrogenase-to-lymphocyte ratio.

Using X-tile, the optimal cutoff values for the LLR, NLR, and PLR were determined to be 471.5, 5.8, and 261.3, respectively. These values are illustrated as black and white circles positioned on the x-axis in Figure 1A, as well as in Figure S1A and 1B, Supplemental Digital Content 1. Figure 1B and Figure S1C and 1D, Supplemental Digital Content 1 depict the frequency of categories that are either below or above the ideal threshold in the histograms. In this study, most patients had a low LLR, NLR, and PLR. Patients were categorized into 2 groups based on the optimal cutoff values for the LLR, NLR, and PLR, and their OS was analyzed using the Kaplan–Meier method (Figure 1C, Figure S1E–F, Supplemental Digital Content 1). Patients in the high LLR and NLR groups exhibited poorer ECOG PS and a higher incidence of liver metastases than those in the low LLR and NLR groups (Table 1, Table S1, Supplemental Digital Content 2). There were no significant differences in the patient characteristics between the high- and low-PLR groups (Table S2, Supplemental Digital Content 3).

### 3.3. OS-related factors: univariate and multivariate analysis

**Figure 2.**
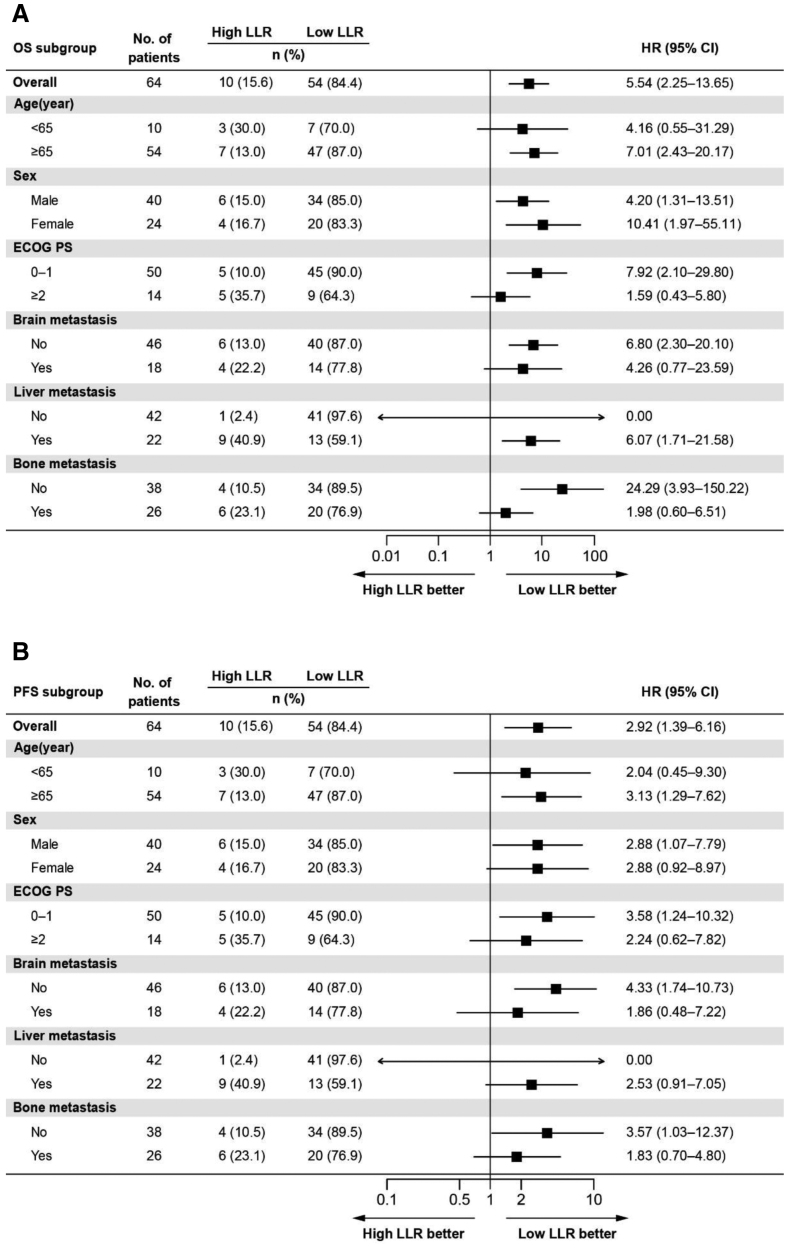
Subgroup analysis of the association between the LLR and OS (A), and PFS (B). CI = confidential interval, ECOG PS = Eastern Cooperative Oncology Group performance status, = HR, hazard ratio, LLR = lactate dehydrogenase-lymphocyte ratio, OS = overall survival, PFS = progression-free survival.

The median duration of observation was 10.5 months (interquartile range: 6.0–19.0 months). By the time of data cutoff, 36 patients (56.3%) had died. Table 2 presents the results of both univariate and multivariate analyses of the factors associated with OS, including the LLR. In the univariate Cox proportional hazards analysis, the ECOG PS, bone metastasis, and LLR were significant (*P* < .05). The multivariate analysis using these factors showed that a high LLR was significantly linked to decreased OS (HR: 3.41; 95% CI: 1.23–9.47, *P* = .02). We examined the association between the LLR and OS within subgroups based on initial patient characteristics. A consistent pattern emerged, indicating that a high LLR was associated with a poor OS (Fig. 2A). We conducted the same analysis for the NLR and PLR. The multivariate analysis revealed that an elevated NLR was significantly linked to a decreased OS (HR: 4.99; 95% CI: 2.26–10.99; *P* < .0001) (Table S3, Supplemental Digital Content 4). However, a high PLR was not associated with OS (Table S4, Supplemental Digital Content 5).

**Table 2:** Results of the univariate and multivariate analyses of factors affecting overall survival (n = 64).

Variables	Category	Univariate analysis			Multivariate analysis		
		HR	95% CI	*P*-value	HR	95% CI	*P*-value
Age (years)	≥65 vs <65	0.86	0.30–2.51	.79	–	–	–
Sex	Female vs Male	1.05	0.53–2.09	.89	–	–	–
ECOG PS	≥2 vs 0–1	2.34	1.10–4.97	.03	1.66	0.73–3.77	.22
Brain metastasis	Yes vs No	0.92	0.45–1.90	.83	–	–	–
Liver metastasis	Yes vs No	2.00	0.99–4.03	.053	–	–	–
Bone metastasis	Yes vs No	2.18	1.11–4.28	.02	1.72	0.83–3.56	.15
LLR	High vs Low	5.54	2.25–13.7	.0002	3.41	1.23–9.47	.02

CI = confidence interval, ECOG PS = Eastern Cooperative Oncology Group performance status, HR = hazard ratio, LLR = lactate dehydrogenase-to-lymphocyte ratio.

### 3.4. PFS-related factors: univariate and multivariate analyses

By the time of data cutoff, 56 patients (87.5%) had experienced PD. Table 3 presents the results of both the univariate and multivariate analyses of the factors associated with PFS, including the LLR. In the univariate Cox proportional hazards analysis, bone metastasis and the LLR were identified as significant factors (*P* < .05). The multivariate analysis using these factors indicated that a high LLR was significantly linked to decreased PFS (HR: 2.38; 95% CI: 1.10–5.15; *P* = .03). We examined the association between the LLR and PFS across different subgroups based on initial patient characteristics. A consistent pattern emerged, indicating that a high LLR was predictive of poor PFS (Fig. 2B). We conducted a similar analysis for the NLR and PLR; however, neither a high NLR nor a high PLR was an independent predictor of PFS (Table S5, Supplemental Digital Content 6, Table S6, Supplemental Digital Content 7).

**Table 3:** Results of the univariate and multivariate analyses of factors affecting progression-free survival (n = 64).

Variables	Category	Univariate analysis			Multivariate analysis		
		HR	95% CI	*P*-value	HR	95% CI	*P*-value
Age (years)	≥65 vs <65	0.62	0.31–1.25	.18	–	–	–
Sex	Female vs Male	0.98	0.57–1.68	.93	–	–	–
ECOG PS	≥2 vs 0–1	1.07	0.55–2.07	.84	–	–	–
Brain metastasis	Yes vs No	0.83	0.46–1.51	.54	–	–	–
Liver metastasis	Yes vs No	1.65	0.96–2.84	.07	–	–	–
Bone metastasis	Yes vs No	2.00	1.15–3.47	.01	1.75	0.98–3.11	.06
LLR	High vs Low	2.92	1.39–6.16	.005	2.38	1.10–5.15	.03

CI = confidence interval, ECOG PS = Eastern Cooperative Oncology Group performance status, HR = hazard ratio, LLR = lactate dehydrogenase-to-lymphocyte ratio.

### 3.5. OS and PFS for all patients and groups based on the LLR

**Figure 3.**
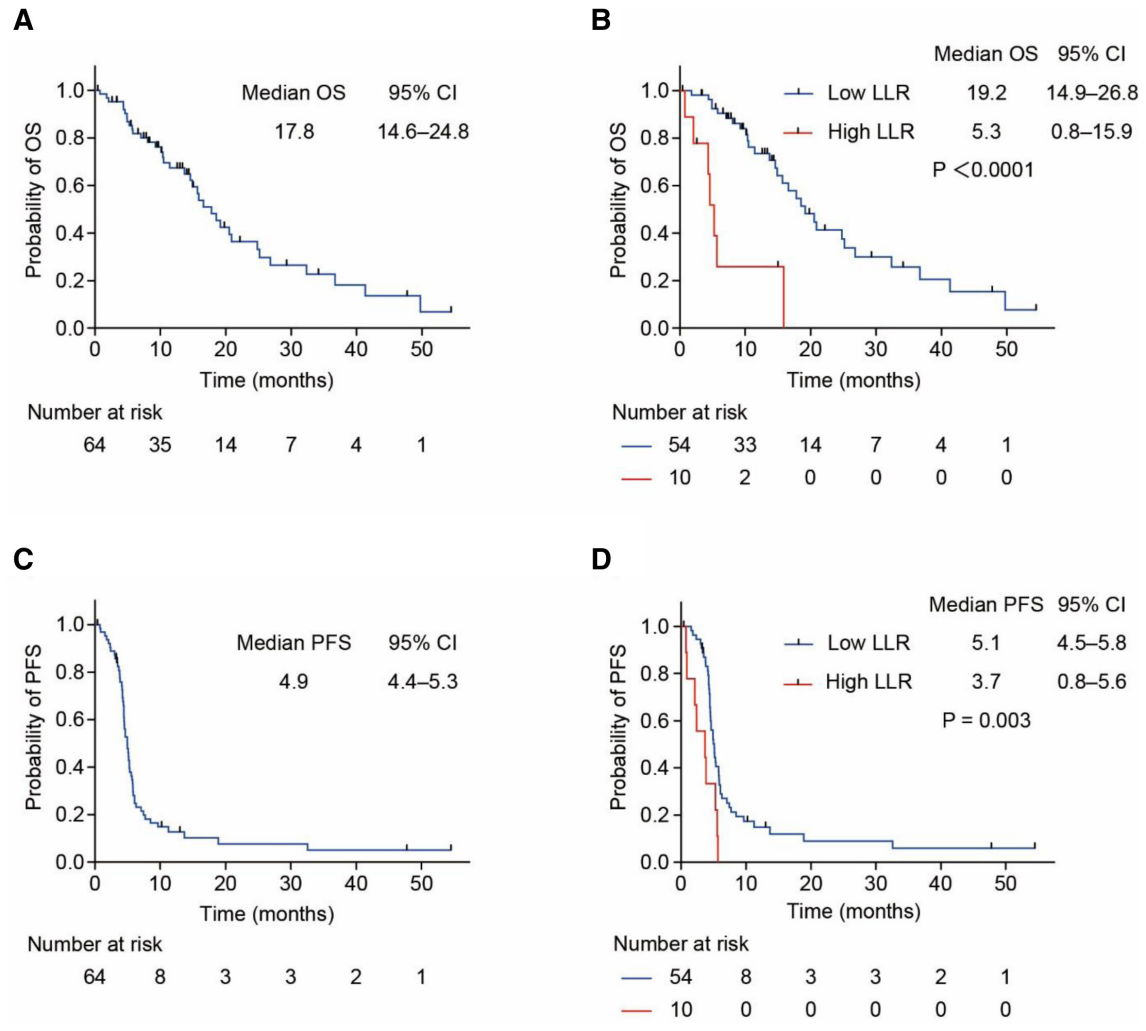
OS and PFS for all patients and subgroups by the LLR. (A) Kaplan–Meier survival curves of the OS for all patients. (B) Kaplan–Meier survival curves of the OS of patients with a low or high LLR. (C) Kaplan–Meier survival curves of the PFS for all patients. (D) Kaplan–Meier survival curves of the PFS of patients with low or high LLR. CI = confidential interval, LLR = lactate dehydrogenase-to-lymphocyte ratio, OS = overall survival, PFS = progression-free survival.

The overall median OS for all patients was 17.8 months (95% CI: 14.6–24.8) (Fig. 3A). Patients with low LLR had a median OS of 19.2 months (95% CI: 14.9–26.8), while those with high LLR had a median OS of 5.3 months (95% CI: 0.8–15.9) (Fig. 3B). The 2 groups showed a statistically significant difference in OS (*P* < .0001). The median PFS for the entire cohort was 4.9 months (95% CI: 4.4–5.3). (Fig. 3C). Patients with low LLR had a median PFS of 5.1 months (95% CI: 4.5–5.8), whereas those with high LLR had a median PFS of 3.7 months (95% CI: 0.8–5.6) (Fig. 3D); hence, a significant difference in PFS was observed between the 2 groups (*P* = .003).

### 3.6. Response to first-line treatment

Table 4 presents the responses to initial treatment. The overall response rate (ORR) and disease control rates (DCRs) for all the participants were 64.1% and 89.1%, respectively. The DCR variation between the low- and high-LLR groups did not reach statistical significance, with values of 68.5% and 40.0%, respectively (*P* = .15). The difference in DCRs between the 2 groups was not statistically significant, with rates of 92.6% and 70.0%, respectively (*P* = .07).

**Table 4:** Best overall response with first-line therapy stratified by the LLR.

Response	Total (n = 64)	Low LLR (n = 54)	High LLR (n = 10)	*P*-value
Complete response (%)	3.1	3.7	0	
Partial response (%)	60.9	64.8	40.0	
Stable disease (%)	25.0	24.1	30.0	
Progressive disease (%)	0	0	0	
Not evaluable (%)	10.9	7.4	30.0	
Objective response rate (%)	64.1	68.5	40.0	.15
Disease control rate (%)	89.1	92.6	70.0	.07

LLR = lactate dehydrogenase to lymphocyte ratio.

### 3.7. Adverse events due to first-line therapy

Table 5 presents the side effects associated with first-line treatment. Over 60% of patients in both the low- and high-LLR categories experienced grade 3 or 4 neutropenia, whereas more than 20% of patients in each group developed febrile neutropenia. In the low LLR group, immune-related adverse events included grade 1 or 2 thyroid dysfunction in 4 patients (7.4%), adrenal insufficiency in 2 patients (3.7%), and arthritis in 1 patient (1.9%). Additionally, grade 3 pneumonitis was noted in 1 patient (1.9%) in the low LLR group and 1 patient (10.0%) in the high LLR group.

**Table 5:** Adverse events with first-line therapy stratified by LLR.

Event	Low LLR group (n = 54)		No. of patients (%)	High LLR group (n = 10)	
	Grade 1 or 2	Grade 3 or 4		Grade 1 or 2	Grade 3 or 4
Any adverse event	33 (61.1)	36 (66.7)		5 (50.0)	7 (70.0)
Neutropenia	4 (7.4)	35 (64.8)		2 (20.0)	6 (60.0)
Febrile neutropenia	0	14 (25.9)		0	2 (20.0)
Thrombocytopenia	11 (20.4)	6 (11.1)		0	2 (20.0)
Anemia	7 (13.0)	5 (9.3)		1 (10.0)	2 (20.0)
Nausea	6 (11.1)	0		0	1 (10.0)
Fatigue	6 (11.1)	0		0	0
Decreased appetite	5 (9.3)	0		1 (10.0)	1 (10.0)
Constipation	3 (5.6)	0		0	0
Liver dysfunction	3 (5.6)	0		0	0
Hiccups	2 (3.7)	0		0	0
Increased creatinine	2 (3.7)	0		0	0
Hypothyroidism	2 (3.7)	0		0	0
Hyperthyroidism	2 (3.7)	0		0	0
Adrenal insufficiency	2 (3.7)	0		0	0
Arthritis	1 (1.9)	0		0	0
Rash	1 (1.9)	0		0	0
Pneumonitis	0	1 (1.9)		0	1 (10.0)
Tumor lysis syndrome	0	1 (1.9)		0	1 (10.0)
Sepsis	0	0		0	1 (10.0)

LLR = lactate dehydrogenase to lymphocyte ratio.

## 4. Discussion

This study demonstrated that a high LLR is an independent poor prognostic factor for both OS and PFS in patients with SCLC treated with first-line platinum plus etoposide plus a PD-L1 inhibitor. Patients with a high LLR exhibited significantly shorter OS and PFS, and multivariate analysis confirmed that a high LLR remained a significant predictor of poor prognosis, even after adjusting for other clinical variables. In contrast, the NLR was an independent prognostic factor for OS but not for PFS, and the PLR was not associated with either OS or PFS. Unlike the NLR and PLR, which primarily reflect host immune status, the LLR is thought to incorporate both immune status and tumor-intrinsic metabolic activity, potentially contributing to its superior prognostic ability.^[20]^

Some of our findings are consistent with those of previous reports on prognostic biomarkers in SCLC. A high NLR before treatment has been reported as a poor prognostic factor for OS in patients with SCLC.^[21]^ Similarly, LDH elevation has long been recognized as a surrogate marker for tumor burden and is associated with worse survival outcomes in SCLC.^[20]^ LDH is a key enzyme involved in anaerobic glycolysis and plays a central role in the Warburg effect, in which cancer cells preferentially utilize glycolysis over oxidative phosphorylation, even in the presence of oxygen. This metabolic shift facilitates rapid adenosine triphosphate production and biosynthesis, supporting tumor growth, invasion, and metastasis.^[22]^ Moreover, the lung immune prognostic index (LIPI), which incorporates LDH and the NLR, has been reported to stratify survival outcomes in patients with advanced lung cancer, and a recent study demonstrated that the LIPI is also predictive of poor prognosis in SCLC.^[23]^ Hence, the LLR may represent a promising novel biomarker that integrates both inflammatory and metabolic aspects of the tumor and host response. The LLR has also been investigated in other malignancies and has been identified as an independent prognostic factor in various cancers. For instance, in diffuse large B-cell lymphoma, renal cell carcinoma, and primary central nervous system lymphoma, a high LLR has been shown to correlate with reduced survival.^[15,16,24]^ Furthermore, meta-analyses, which selected a total of 129 articles involving 18,780 cases, have confirmed that a high pretreatment NLR is associated with poor survival in patients with various carcinomas undergoing immunotherapy, suggesting a broader applicability of immune-metabolic biomarkers like LLR.^[25]^ The consistency of our findings with these reports supports the clinical relevance of the LLR as a prognostic biomarker in ES-SCLC.

The potential clinical significance of our research is that the LLR may help identify patients who require closer follow-up after the initiation of first-line therapy. Hence, it may be possible to appropriately connect patients with a high LLR before primary treatment to the next treatment option by shortening the follow-up evaluation interval. The LLR can be easily obtained from routine blood tests and may be used as a cost-effective biomarker.

This study has a few limitations. First, this was a retrospective study with a relatively small number of participants from 2 university hospitals in Japan, which may limit the generalizability of our findings. In particular, only 10 patients were classified into the high-LLR group, and the LLR values in this group showed considerable dispersion. Therefore, the survival estimates from the Kaplan–Meier analysis and the hazard ratios from the multivariate Cox proportional hazards model should be interpreted with caution because of the limited statistical power and potentially wide uncertainty around the estimates. Accordingly, this study should be regarded as exploratory and hypothesis-generating. Further validation in larger multicenter cohorts is necessary to confirm the robustness of the LLR as a prognostic biomarker. Second, the optimal LLR cutoff value identified in this study may not be universally applicable to other patient populations; hence, additional studies are required to determine standardized cutoff values. Third, potential confounding factors, including unmeasured immune-related variables, may have influenced our results, necessitating a cautious interpretation of our findings.

To establish the prognostic value of the LLR in SCLC, prospective validation in larger, multicenter studies is warranted. A prospective clinical trial assessing the LLR in a broader cohort of patients with SCLC treated with chemoimmunotherapy may provide more definitive evidence for its utility. Future studies should also explore the dynamic changes in the LLR during treatment, as temporal variations in the LLR may provide additional prognostic information beyond baseline measurements. Moreover, incorporating the LLR into a comprehensive prognostic model alongside other biomarkers, such as PD-L1 expression and tumor mutational burden, could enhance predictive accuracy. Given the limitations of single biomarkers, a multiparametric approach combining the LLR with additional immune and metabolic markers may be necessary to optimize SCLC prognosis.^[26]^

In summary, this exploratory study suggests that the LLR may be a potential prognostic biomarker for OS and PFS in patients with ES-SCLC who underwent initial chemoimmunotherapy. Compared with the NLR and PLR, the LLR may better reflect both tumor metabolism and the host immune response, making it a more comprehensive prognostic indicator. However, further prospective validation in larger cohorts is needed before the LLR can be applied as a standalone risk stratification tool in clinical practice.

## Acknowledgments

We would like to thank Editage (www.editage.com) for the English language editing.

## Author contributions

**Conceptualization:** Tomofumi Hirose, Shuhei Teranishi.

**Writing – review & editing:** Nobuaki Kobayashi, Masaki Yamamoto, Makoto Kudo, Takeshi Kaneko.

**Investigation:** Anna Tanaka, Yukihito Kajita, Ayami Kaneko.














