# Supplementary material for: Lactate dehydrogenase-to-lymphocyte ratio predicts overall and progression-free survival among patients with advanced small-cell lung cancer receiving first-line chemoimmunotherapy: A two-center retrospective observational study
Source: Medicine (Baltimore). 2026 Aug 14;105(33):e50249. doi: 10.1097/MD.0000000000050249 (PMC13480915; doi:10.1097/MD.0000000000050249)
Supplement: Supplementary file 1 [file medi-105-e50249-s002.docx]

**Supplemental Table 1**. Patient characteristics in the NLR group

| Characteristics | All patients  (n = 64) | Low NLR  (n = 52) | High NLR  (n = 12) | P-value |
| --- | --- | --- | --- | --- |
| Age (years) | 73 (66–76) | 72 (66–76) | 75 (72–80) | .08 |
| <65 years | 10 (15.6) | 9 (17.3) | 1 (8.3) |  |
| ≥65 years | 54 (84.4) | 43 (82.7) | 11 (91.7) |  |
| Sex |  |  |  | .75 |
| Male | 40 (62.5) | 33 (63.5) | 7 (58.3) |  |
| Female | 24 (37.5) | 19 (36.5) | 5 (41.7) |  |
| Smoking status |  |  |  | > .99 |
| Never | 3 (4.7) | 3 (5.8) | 0 |  |
| Current or former | 61 (95.3) | 49 (94.2) | 12 (100.0) |  |
| ECOG PS |  |  |  | .006 |
| 0 | 12 (18.8) | 12 (23.1) | 0 |  |
| 1 | 38 (59.4) | 33 (63.5) | 5 (41.7) |  |
| 2 | 10 (15.6) | 5 (9.6) | 5 (41.7) |  |
| 3 | 4 (6.3) | 2 (3.9) | 2 (16.7) |  |
| 4 | 0 | 0 | 0 |  |
| Stage |  |  |  | > .99 |
| Post-treatment recurrence of LS | 7 (10.9) | 6 (11.5) | 1 (8.3) |  |
| ES | 57 (89.1) | 46 (88.5) | 11 (91.7) |  |
| Programmed death-ligand 1 inhibitors |  |  |  | .73 |
| Atezolizumab | 42 (65.6) | 35 (67.3) | 7 (58.3) |  |
| Durvalumab | 22 (34.4) | 17 (32.7) | 5 (41.7) |  |
| Brain metastasis |  |  |  | > .99 |
| No | 46 (71.9) | 37 (71.2) | 9 (75.0) |  |
| Yes | 18 (28.1) | 15 (28.9) | 3 (25.0) |  |
| Liver metastasis |  |  |  | .02 |
| No | 42 (65.6) | 38 (73.1) | 4 (33.3) |  |
| Yes | 22 (34.4) | 14 (26.9) | 8 (66.7) |  |
| Bone metastasis |  |  |  | > .99 |
| No | 38 (59.4) | 31 (59.6) | 7 (58.3) |  |
| Yes | 26 (40.6) | 21 (40.4) | 5 (41.7) |  |
| NLR | 3.4 (2.6–5.3) | 3.2 (2.6–4.1) | 7.6 (6.4–8.8) | < .0001 |
| Low NLR (≤5.8) | 52 (81.3) | 52 (100.0) | 0 |  |
| High NLR (>5.8) | 12 (18.8) | 0 | 12 (100.0) |  |

Data are presented as numbers (%) or medians (interquartile ranges). Fisher's exact test was used to compare the proportions of categorical data between the groups. The Mann–Whitney *U* test was used to compare numerical data between the groups. ECOG PS, Eastern Cooperative Oncology Group Performance Status; ES, extensive-stage; IQR, interquartile range; LS, limited stage; NLR, neutrophil-to-lymphocyte ratio.
