# Supplementary material for: Lactate dehydrogenase-to-lymphocyte ratio predicts overall and progression-free survival among patients with advanced small-cell lung cancer receiving first-line chemoimmunotherapy: A two-center retrospective observational study
Source: Medicine (Baltimore). 2026 Aug 14;105(33):e50249. doi: 10.1097/MD.0000000000050249 (PMC13480915; doi:10.1097/MD.0000000000050249)
Supplement: Supplementary file 2 [file medi-105-e50249-s003.docx]

**Supplemental Table 2**. Patient characteristics in the PLR group

| Characteristics | All patients (n = 64) | Low PLR  (n = 46) | High PLR  (n = 18) | P-value |
| --- | --- | --- | --- | --- |
| Age (years) | 73 (66–76) | 74 (66–76) | 72 (66–75) | .48 |
| <65 years | 10 (15.6) | 8 (17.4) | 2 (11.1) |  |
| ≥65 years | 54 (84.4) | 38 (82.6) | 16 (88.9) |  |
| Sex |  |  |  | .09 |
| Male | 40 (62.5) | 32 (69.6) | 8 (44.4) |  |
| Female | 24 (37.5) | 14 (30.4) | 10 (55.6) |  |
| Smoking status |  |  |  | 1.00 |
| Never | 3 (4.7) | 2 (4.4) | 1 (5.6) |  |
| Current or former | 61 (95.3) | 44 (95.7) | 17 (94.4) |  |
| ECOG PS |  |  |  | .71 |
| 0 | 12 (18.8) | 10 (21.7) | 2 (11.1) |  |
| 1 | 38 (59.4) | 27 (58.7) | 11 (61.1) |  |
| 2 | 10 (15.6) | 6 (13.0) | 4 (22.2) |  |
| 3 | 4 (6.3) | 3 (6.5) | 1 (5.6) |  |
| 4 | 0 | 0 | 0 |  |
| Stage |  |  |  | .18 |
| Post-treatment recurrence of LS | 7 (10.9) | 7 (15.2) | 0 |  |
| ES | 57 (89.1) | 39 (84.8) | 18 (100.0) |  |
| Programmed death-ligand 1 inhibitors |  |  |  | .08 |
| Atezolizumab | 42 (65.6) | 27 (58.7) | 15 (83.3) |  |
| Durvalumab | 22 (34.4) | 19 (41.3) | 3 (16.7) |  |
| Brain metastasis |  |  |  | .35 |
| No | 46 (71.9) | 35 (76.1) | 11 (61.1) |  |
| Yes | 18 (28.1) | 11 (23.9) | 7 (38.9) |  |
| Liver metastasis |  |  |  | .38 |
| No | 42 (65.6) | 32 (69.6) | 10 (55.6) |  |
| Yes | 22 (34.4) | 14 (30.4) | 8 (44.4) |  |
| Bone metastasis |  |  |  | .78 |
| No | 38 (59.4) | 28 (60.9) | 10 (55.6) |  |
| Yes | 26 (40.6) | 18 (39.1) | 8 (44.4) |  |
| PLR | 208.7 (163.2–292.9) | 185.8 (142.0–214.0) | 329.8 (306.4–413.2) | < .0001 |
| Low PLR (≤261.3) | 46 (71.9) | 46 (100.0) | 0 |  |
| High PLR (>261.3) | 18 (28.1) | 0 | 18 (100.0) |  |

Data are presented as numbers (%) or medians (interquartile ranges). Fisher's exact test was used to compare the proportions of categorical data between the groups. The Mann–Whitney *U* test was used to compare numerical data between the groups. ECOG PS, Eastern Cooperative Oncology Group Performance Status; ES, extensive stage; IQR, interquartile range; LS, limited stage; PLR, platelet-lymphocyte ratio.
