# Supplementary material for: Lactate dehydrogenase-to-lymphocyte ratio predicts overall and progression-free survival among patients with advanced small-cell lung cancer receiving first-line chemoimmunotherapy: A two-center retrospective observational study
Source: Medicine (Baltimore). 2026 Aug 14;105(33):e50249. doi: 10.1097/MD.0000000000050249 (PMC13480915; doi:10.1097/MD.0000000000050249)
Supplement: Supplementary file 3 [file medi-105-e50249-s004.docx]

**Supplemental Table 3**. Univariate and multivariate analyses of factors affecting overall survival including the NLR (n = 64)

| Variables | Category | Univariate analysis | | |  | Multivariate analysis | | |
| --- | --- | --- | --- | --- | --- | --- | --- | --- |
|  |  | HR | 95% CI | P*-*value |  | HR | 95% CI | P*-*value |
| Age (years) | ≥65 vs. <65 | 0.86 | 0.30–2.51 | .79 |  | – | – | – |
| Sex | Female vs. Male | 1.05 | 0.53–2.09 | .89 |  | – | – | – |
| ECOG PS | ≥2 vs. 0–1 | 2.34 | 1.10–4.97 | .03 |  | 1.06 | 0.53–2.15 | .87 |
| Brain metastasis | Yes vs. No | 0.92 | 0.45–1.90 | .83 |  | – | – | – |
| Liver metastasis | Yes vs. No | 2.00 | 0.99–4.03 | .053 |  | – | – | – |
| Bone metastasis | Yes vs. No | 2.18 | 1.11–4.28 | .02 |  | 1.28 | 0.72–2.26 | .41 |
| NLR | High vs. Low | 5.38 | 2.12–13.7 | .0004 |  | 4.99 | 2.26–10.99 | < .0001 |

CI, confidence interval; ECOG PS, Eastern Cooperative Oncology Group performance status; HR, hazard ratio; NLR, neutrophil-lymphocyte ratio.
