# Supplementary material for: Lactate dehydrogenase-to-lymphocyte ratio predicts overall and progression-free survival among patients with advanced small-cell lung cancer receiving first-line chemoimmunotherapy: A two-center retrospective observational study
Source: Medicine (Baltimore). 2026 Aug 14;105(33):e50249. doi: 10.1097/MD.0000000000050249 (PMC13480915; doi:10.1097/MD.0000000000050249)
Supplement: Supplementary file 5 [file medi-105-e50249-s006.docx]

**Supplemental Table 5**. Univariate and multivariate analyses of the factors affecting progression-free survival including the NLR (n = 64)

| Variables | Category | Univariate analysis | | |  | Multivariate analysis | | |
| --- | --- | --- | --- | --- | --- | --- | --- | --- |
|  |  | HR | 95% CI | P*-*value |  | HR | 95% CI | P*-*value |
| Age (years) | ≥65 vs. <65 | 0.62 | 0.31–1.25 | .18 |  | – | – | – |
| Sex | Female vs. Male | 0.98 | 0.57–1.68 | .93 |  | – | – | – |
| ECOG PS | ≥2 vs. 0–1 | 1.07 | 0.55–2.07 | .84 |  | – | – | – |
| Brain metastasis | Yes vs. No | 0.83 | 0.46–1.51 | .54 |  | – | – | – |
| Liver metastasis | Yes vs. No | 1.65 | 0.96–2.84 | .07 |  | – | – | – |
| Bone metastasis | Yes vs. No | 2.00 | 1.15–3.47 | .01 |  | 1.82 | 1.03–3.19 | .04 |
| NLR | High vs. Low | 2.10 | 1.08–4.10 | .03 |  | 1.79 | 0.90–3.55 | .10 |

CI, confidence interval; ECOG PS, Eastern Cooperative Oncology Group performance status; HR, hazard ratio; NLR, neutrophil-lymphocyte ratio.
